# Yes: The Symptoms of OCD and Depression Are Discrete and Not Exclusively Negative Affectivity

**DOI:** 10.3389/fpsyg.2017.00753

**Published:** 2017-05-12

**Authors:** Kathleen A. Moore, Jacqui Howell

**Affiliations:** School of Health Sciences and Psychology, Federation University AustraliaChurchill, VIC, Australia

**Keywords:** obsessive-compulsive disorder, depression, negative affectivity, discrete disorders, relationship

## Abstract

Although Obsessive-Compulsive Disorder (OCD) and Depression are classified as separate disorders, the high incidence of co-morbidity and the strong correlations between measures of each has led to debate about the nature of their relationship. Some authors have proposed that OCD is in fact a mood disorder while others have suggested that the two disorders are grounded in negative affectivity. A third proposition is that depression is an essential part of OCD but that OCD is a separate disorder from depression. The aim in this study was to investigate these diverse propositions in a non-clinical sample and also to determine whether factors implicated in each, that is anxious and depressive cognitions, hopelessness, and self-criticism, would demonstrate commonality as predictors of the symptoms of OCD and of depression. Two hundred participants (59% female) (*M* age = 34 years, *SD* = 16) completed the Padua Inventory, Carroll Rating Scale, Cognitions Checklist, Self-Criticism Scale, Beck Hopelessness Scale, Buss-Durkee Hostility Inventory-Revised and a Negative Affectivity Schedule. Results indicated a strong correlation between OCD and depression, depression, and negative affectivity but a weaker relationship between OCD and negative affectivity. Path analyses revealed that both anxious and depressive cognitions, as well as hostility predicted both disorders but the Beta-weights were stronger on OCD. Self-criticism predicted only depression while hopelessness failed to predict either disorder but was itself predicted by depressive cognitions. Depression was a stronger indicator of negative affect than OCD and while OCD positively predicted depression, depression was a negative indicator of OCD. These results support the hypothesis that OCD and depression are discrete disorders and indicate that while depression is implicated in OCD, the reverse does not hold. While both disorders are related to negative affectivity, this relationship is much stronger for depression thus failing to confirm that both are subsumed by a common factor, in this case, negative affectivity. The proposition that depression is part of OCD but that OCD is not necessarily implicated in depression and is, in fact, a separate disorder, is supported by the current model. Further research is required to support the utility of the model in clinical samples.

## Introduction

Obsessive Compulsive Disorder (OCD) and Major Depressive Disorder (MDD) are both personally, socially and occupationally incapacitating. Historically, both OCD and MDD were classified as psychoneurotic disorders in DSM-1 (American Psychiatric Association, 1952) and it was not until DSM-111 (American Psychiatric Association, 1980) that a clear distinction was made between them. Despite the ongoing diagnostic separation, the incidence of co-morbidity is high with most studies suggesting that at least one third of OCD sufferers have concurrent depression at the time of assessment (Nestadt et al., 2001; Abramowitz, 2004; Hong et al., 2004) and in cases of severe depression, anxiety disorders including obsessive compulsive symptoms are often present (Brown and Barlow, 1992; Kessler et al., 2003). The high levels of comorbidity between the disorders has lead researchers such as Kessler et al. (2003) to argue that this relationship does not occur by chance or ascertainment bias alone. In an earlier study, Tiller (1994) suggested that the high incidence of co-morbidity between the disorders is because OCD is in fact a mood disorder, whereas Billet et al. (1998) and later Goes et al. (2012) suggested that they might share a common genetic diathesis. Somewhat aligned with this suggestion is Brown et al.'s (1997) proposition that both may be grounded in negative affectivity. However others, for example, Freeman (1992) and Montgomery (1993) while acknowledging the high co-morbidity between the disorders, consider that OCD and MDD are discrete conditions. These propositions are both intriguing and unresolved. It is the aim of the present study to investigate these hypotheses by examining the relationship between OCD and MDD, the possible shared predictors of each, and the relationship between OCD and MDD with negativity affectivity. As significant numbers of people experience each disorder and, when they are comorbid, the impact is greater, it is important for clinicians to have a comprehensive understanding of these inter-relationships to inform treatments, particularly those of a psychological nature.

Substantial overlap between OCD and MDD can also be seen at the clinical level. In OCD, people with checking compulsions may *fear* (an emotion commonly associated with anxiety) that they have run over someone while driving, yet may also feel a sense of *hopelessness* (a common symptom of depression) associated with the failure of their checking to reassure them that no one had been run over. Depressed people also experience high levels of anxious symptoms such as agitation, apprehension and worry. Clark et al. (1994), Kiloh and Garside (1963), and Levitt and Lubin (1975) suggested that overt anxiety is commonly found in reactive depression but is infrequently observed in the more severe psychotic or bipolar forms of depression. It is in the more severe forms of depression that obsessions and compulsions are seen which is also indicated by scales such as the Hamilton Observer Rating Scale for Depression (Hamilton, 1960) where these symptoms are included in supplementary items to assess the severity of depression.

Some researchers (e.g., Pallanti et al., 2011) have suggested that OCD, like depression, is heterogeneous in nature which, if so, can be argued to support their uniqueness. Support for this premise is seen in the earlier work of Ricciardi and McNally (1995) who found that depressive symptoms were often more strongly related with the obsessive rather than compulsive symptoms of OCD. Similarly, Quarantini et al. (2011) found depression was associated with just four of six OC dimensions they investigated. Despite this variability, the high levels of comorbidity found in clinical populations and the moderate to strong inter-correlations between measures of MDD and OCD (van Oppen et al., 1995; Taylor, 1998; Yap et al., 2012) suggest a commonality.

The strong association between measures of anxiety and depression is well documented, with co-efficients typically in the range of 0.50–0.80 (Gotlib, 1983; Nezu et al., 1986; Zurawski and Smith, 1987) which may have prompted Kendall and Watson (1990) to suggest that anxiety and depression reflect a single undifferentiated factor. Support for this claim comes from factor analytic studies which have shown that the two disorders are dominated by a second-order factor called *negative affectivity* (Gotlib, 1983; Watson and Clark, 1984; Zurawski and Smith, 1987; Watson et al., 1988a), often referred to as neuroticism or general distress (Kendall and Watson, 1990). Although, their study was limited to general measures of anxiety and depression in a sample of children, Lonigan et al. (2003) found that scores on these scales correlated significantly with negative affectivity.

High negative affect is composed of a wide range of factors reflecting *fear, nervousness, anger, guilt, hostility, sadness, loneliness, self-criticism*, and *self-dissatisfaction*, while low negative affect is best defined as calm and relaxed. These negative terms describe aspects of both OCD and depression (Kendall and Watson, 1990). Negative affectivity is highly convergent with self-report ratings of mood. For example, Watson et al. (1988b) used the Hopkins Symptom Checklist (HSCL) (Derogatis et al., 1974), a measure of the degree of distress associated with a variety of depressive and anxious symptoms in a sample of undergraduates, and found that trait negative affectivity was related to a broad array of complaints, including all symptoms of OCD and depression. Negative affectivity was also significantly related to all four subscale scores of the HSCL: general anxiety, OCD, somatization and depressive disorder.

Cognitive frameworks for MDD are well established (Beck, 1967, 1976; Alloy et al., 1985) with authors such as Beck proposing a triad of depressive symptoms. Depressed patients score higher on measures of negative thinking about the self, their current situation and the future (Lam et al., 1987; McCauley et al., 1988), abandonment and loss (Parrish and Radomsky, 2010) than non-depressed control participants (Crandell and Chambless, 1986; Dobson and Shaw, 1986; Dohr et al., 1989), non-psychiatric medical patients (Harrell and Ryan, 1983; Hollon et al., 1986) and themselves when the condition has remitted (Eaves and Rush, 1984; Dobson and Shaw, 1986; Dohr et al., 1989). Ostrander et al. (1995) argued that negative self-cognitions are not specific to depression as they found that depressogenic cognitions, including negative cognitive errors and hopelessness, were also associated with OCD. Also Van Den Hout and Hessels (1984) found that lowered mood in patients with OCD was associated with self-denigrating thoughts (e.g., I am ridiculous) and a sense of hopelessness (e.g., my future offers dismal prospects if I continue this). In fact, Clark (2002) asked whether there are any cognitive processes in OCD that are unique from those in depression albeit mentioning the stronger correlations typically found between OCD, responsibility and perfectionism.

Another component of Beck's theory relates to self-schemas: the ways in which we perceive ourselves and the world around us. A pervasive negative bias among depressed individuals reflects their greater efficiency in processing negative personal semantic information compared to control groups (Kuiper and Derry, 1982; Hammen et al., 1986). Depressed individuals also show a lack of ability to process (Ingram et al., 1983) and encode positive personal information given to them (Roth and Rehm, 1980; Gotlib, 1981). Relatively little has been written about specific information processing bias in OCD patients. However, the notion that a cognitive mode characterized by threat and danger to one's personal domain or others is, according to Salkovskis (1985,1989), valid. The cognitive threat mode results in OCD patients being hypervigilant to stimuli which signal danger or threat. For example, OCD sufferers may focus on the dangerous aspects of a potentially contamination causing object, while minimizing or ignoring *safety* features such as washing oneself after coming in contact with the object. In this way, the threat inducing cognitive mode is associated with individuals' negative self-appraisal, in that an exaggerated sense of vulnerability is experienced which leads to individuals minimizing their ability to cope with the situation.

Authors such as Steketee (1993) have found a strong association between a variety of distorted cognitions and OCD leading her to suggest that OCD is fundamentally a disorder of disturbed cognitive processing. Consistent with Steketee's (1993) claims, Salkovskis (1985,1989) also found that cognitive errors, or irrational beliefs, include patients overestimating the risk of negative consequences of their actions, experiencing exaggerated guilt, and having an increased perception of threat. In addition, Taylor et al. (2002) discussed the presence of over generalizations, lack of confidence in memory, and an intolerance for uncertainly and change among the cognitive distortions experienced by people with OCD.

Psychoanalytic theorists were among the first to assert the importance of aggressive impulses in OCD. Support for their theories comes from the proportion of OCD sufferers who report obsessional symptoms with themes of aggression (Levenkron, 1991). These observations are consistent with Moore (2017) who found an association between hostility and the subscales of the Padua Inventory (Senavio, 1988) particularly the loss of motor control subscale. Moore suggested that the pattern of findings was not surprising given the patients concern over losing control of violent/harmful obsessions and worries. Research has also shown links between anger and aggression in people with depression (Busch, 2009) the expression of which can be either inward or outward (Luutonen, 2007).

Kendall (1970) and Selby (1986) found that depression was linked to inward but not outward aggression. In a later study, Moreno et al. (1993), using measures assessing various aspects of hostility, found depression was most strongly associated with intro-punitiveness or inner directed hostility followed by suspicion of others and guilt. Hayworth et al. (1980) disputed this finding while Luutonen (2007) reported that anger and hostility could be expressed in either direction. Despite the debate over directionality, anxiety and depression scales are highly correlated with measures of anger and hostility as well as levels of general maladjustment (Dobson, 1985; Tanaka-Matsumi and Kameoka, 1986).

Self-criticism has been implicated in both depression (Blatt et al., 1995) and OCD (Steketee, 1993). Hewitt and Flett (1990, 1991) found that self-criticism was strongly related to two aspects of perfectionism: self-oriented and socially prescribed perfectionism. Depressed individuals with self-oriented perfectionism desire to be perfect, strive to meet exaggerated and unrealistic standards and focus on their own flaws, failures and shortcomings. Furthermore, people's focus on perceived personal flaws and shortcomings are associated with harsh self-scrutiny, such that when perfectionist demands are not met, these individuals engage in self-criticism (Hewitt and Flett, 1990, 1991). These findings concur with Frost et al. (1991) who also found associations between the self-critical appraisals of patients with OCD and their need to fulfill perfectionist expectations set by self and others to be consistent with Beck's (1967) theory that depressed and OCD patients hold negative views of themselves.

It would be a mistake to conclude that the often poor discriminant findings between depression and OCD simply reflect the limitations of self-report assessments because as Deluty et al. (1986) observed, clinicians and others' ratings of anxiety and depression are strongly related. That people with each condition have shown treatment response to antidepressants (Bolwig et al., 2007; McGrath et al., 2008) in particular, to selective serotonin reuptake inhibitors (SSRIs) such as fluvoxamine (Apter et al., 1994) and fluoxetine (Meltzer et al., 1997) adds to the debate. Although, not a focus of this paper, it is important to note that research has also provided support for the role of the ventromedial prefrontal cortex in both OCD and depression (Cavedini et al., 2002; Myers-Schulz and Koenigs, 2012).

In summary, OCD and depression are two distinct classificatory disorders yet the high levels of comorbidity and strong scalar correlations have prompted some to suggest that OCD is in fact a depressive disorder. In a different approach, Brown et al. (1997) proposed that negative affectivity subsumes both these disorders. These propositions warrant further investigation as understanding the relationship between OCD and depression is important not only for classificatory purposes but also for treatment.

**Figure 1:**
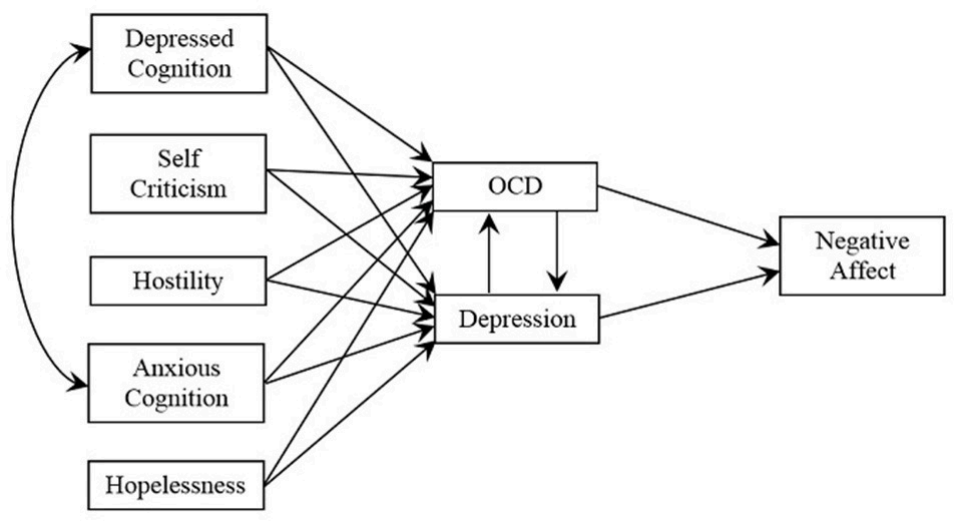
**Hypothesized model of the psychological predictors of OCD and depression, the relationship of OCD with depression and their impact on negative affectivity**.

From the literature it is hypothesized that: the symptoms of OCD and MDD will demonstrate a strong relationship; the symptoms of both OCD and depression will be positively related to negative affectivity and, consistent with a shared negative affectivity factor (Watson and Tellegen, 1985; Kendall and Watson, 1990) the symptoms of both depression and OCD will be positively predicted by self-criticism, hostility, and hopelessness. According to the cognitive content specificity hypothesis (Beck et al., 1987; Clark et al., 1990). It is further hypothesized that anxious and depressive cognitions will be positively related and each will predict scores on both the depression and OCD scales (Figure 1).

## Methods

### Design

A cross-sectional design was used to explore the relationship between MDD and OCD, the commonality of predictors, as well as the contribution of MDD and OCD to negativity affect in a non-clinical sample.

### Participants

Two hundred volunteers (63% females, 37% males) (*M* age = 34 years, *SD* = 16) volunteered to participate in this study. Only 2% of participants reported that suffered from a mental illness at the time of the study. Due to these small, and possibly representative percentages, no attempt was made to statistically manipulate their effect.

### Procedure

The study was approved by the University's Human Ethics Committee to be conducted in accord with the National Health and Medical Research Council Australia (NHMRC) guidelines. Advertisements were placed on University notice boards inviting interested staff and students to participate in a study looking at the relationship between OCD and depression. Participants were provided with a link to an online survey where a Plain Language Statement provided further details of the study, advised readers that their participation was anonymous, and that they could withdraw from the study at any time by closing their web browser. They were also advised that submission of the completed questionnaire would be deemed to be their informed consent.

### Measures

All participants were asked to provide demographic data on age, gender, and health status, and completed the following self-report questionnaires.

The Padua Inventory (PI; Senavio, 1988) revised by Burns et al. (1996) to remove the items related to worry resulted in a 39 item measure of obsessions and compulsions. Items are rated from 0 = *not at all* to 4 = *very much*. Burns et al. reported that the 39 item version correlated 0.74 with the original scale. Internal reliability for the revised version in the current data was strong (α = 0.92).

Carroll Rating Scale (CRS; Carroll et al., 1981) is a 52-item measure of depressive symptoms. Items are answered on a Yes/No format, of which 12 items require reverse coding. Split half reliability is moderate at 0.55; and support for the validity of the CRS is provided through its correlation with the Hamilton Rating Scale (HAMD; Hamilton, 1960) (*r* = 0.80) (Feinberg et al., 1981).

Cognitions Checklist (CCL; Beck et al., 1987) is a 26-item scale with 14 items assessing the frequency of depressive related cognitions and 12 items assessing anxiety related cognitions, rated 0 = *rarely* to 4 = *always occurs*. The CCL has good internal consistency (α = 0.90 for anxiety; α = 0.72 for depressive cognitions), and test re-test stability (*r* = 0.76 over a 6 week period).

Positive and Negative Affectivity Schedule (PANAS; Watson et al., 1988b) is a 20 item measure of positive and negative affectivity. Responses are rated 1 = *very slightly* or not at all to 5 = *extremely* in relation to how the respondent felt over the past week. Internal consistencies are high (α ≥ 0.85). Only the 10-item negative affectivity subscale was used in this study.

Buss-Durkee Hostility Inventory-Revised (BDHS-R; Schill et al., 1990) contains 21 items across two factors: overt and covert hostility. Items are scored on a *true/false* format. Internal consistency is moderate, α = 0.67 and 0.77 for the covert and overt subscales, respectively. Evidence for its construct validity is provided via negative correlations between Spielberger's (1988) anger-control scale (*r* = −0.65 overt hostility; *r* = −0.48 covert hostility), and positive correlations with anger-in (*r* = 0.57) and anger-out (*r* = 0.37).

Beck Hopelessness Scale (BHS; Beck et al., 1974) is a 20-item scale assessing negative expectancy in future life. Items are rated as either *true/false* with 11 items reflecting hopelessness, and nine hopefulness items which are recoded. Glanz et al. (1995) reported high test-re-test correlation (1 month interval *r* = 0.85) and strong internal consistency (KR_20_ = 0.93).

Self-Criticism Scale (SCS; Moore, 2017) is a 32-item scale, with 16 items each assessing self-criticism and criticism of others. Only self-criticism was utilized in the current study. Items are rated as either *true/false*. The SCS has independent factor structure and item-total correlations for self-criticism (0.41 to 0.65) and criticism of others (0.36 to 0.49) which discriminate between respondents.

## Results

Data were analyzed using SPSS and AMOS (Version 22). Data were screened for normality, linearity and homoscedasticity. The intercorrelations among the variables, means, standard deviations and Cronbach's alpha are presented in Table 1.

**Table 1:** **Intercorrelations among the study variables, their means, standard deviations, and alphas**.

	**1**	**2**	**3**	**4**	**5**	**6**	**7**	**8**
1 OCD	1							
2 Depression	0.590^**^	1						
3 Negative affect	0.430^**^	0.545^**^	1					
4 Anxious cognitions	0.514^**^	0.573^**^	0.402^**^	1				
5 Depressed cognitions	0.521^**^	0.684^**^	0.514^**^	0.676^**^	1			
6 Hopelessness	0.268^**^	0.448^**^	0.256^**^	0.276^**^	0.588^**^	1		
7 Hostility	0.408^**^	0.513^**^	0.339^**^	0.344^**^	0.348^**^	0.298^**^	1	
8 Criticism of self	0.413^**^	0.683^**^	0.475^**^	0.476^**^	0.684^**^	0.558^**^	0.486^**^	1
Mean	17.52	8.75	16.89	6.61	7.90	1.67	8.13	4.45
SD	17.00	5.85	6.67	5.98	8.71	2.18	3.50	3.85
Alpha	0.92	0.86	0.82	0.88	0.93	0.77	0.69	0.84

** *p < 0.01*.

Pearson Product-Moment correlations reveal that depression and OCD are significantly correlated (*r* = 0.59). Both OCD and depression are also significantly correlated with negative affectivity, anxious cognitions, depressive cognitions, hopelessness, hostility, and self-criticism.

### Path model of OCD and depression on negative affectivity

In order to test the hypothesized model (Figure 1) path analysis was conducted using AMOS which provides estimates of the magnitude and significance of hypothesized causal relationships among variables. The Independence $\chi_{\left(7\right)}^{2}$ = 798.16, *p* < 0.001 indicated the suitability of the correlation matrix for analysis.

The data failed to provide a good fit to the hypothesized model ($\chi_{\left(11\right)}^{2}$ = 405.39, *p* < 0.001, Table 2). In line with modifications indices considered theoretically relevant (Schumacker and Lomax, 1996), the paths: depressive cognitions to self-criticism, anxious cognitions to hopelessness, and self-criticism to hostility, were added to the model and provided a reasonable fit ($\chi_{\left(11\right)}^{2}$ = 67.99, *p* < 0.001). In order to provide a more parsimonious solution, those paths not significant in the model were removed with the final model being well supported by the data ($\chi_{\left(11\right)}^{2}$ = 37.28 *p* < 0.001; Goodness of Fit 0.958, Normative Fit Index 0.953, Comparative Fit Index 0.968, RMSEA 0.08 and p/close 0.05). The path from anxious cognitions to depression, while not significant, was retained in the model and it was in the borderline range (*p* < 0.01) and also considered theoretically relevant.

**Table 2:** **Fit statistics for hypothesized and final models**.

	**χ^2^**	**df**	***p***	**c/min**	**GFI**	**NFI**	**CFI**	**RMSEA**	**pclose**
**Hypothesized Model**	405.39	11	0.001	36.85	0.592	0.688	0.424	0.161	0.001
**Paths added**	67.99	11	0.001	6.18	0.926	0.915	0.926	0.161	0.001
Depressive cognitions → Self criticism									
Anxious cognitions → Hopelessness									
Self criticism → Hostility									
**Non significant paths removed**	37.28	13	0.001	2.86	0.958	0.953	0.968	0.087	0.05
Hopelessness → OCD									
Hopelessness → Depression									
Self criticism → OCD									

*c/Min, Normed χ^2^; GFI, Goodness of Fit Index; NFI, Normed fit Index; CFI, Comparative Fit Index; RMSEA, Root Mean Square Error of Approximation; p/close*.

**Figure 2:**
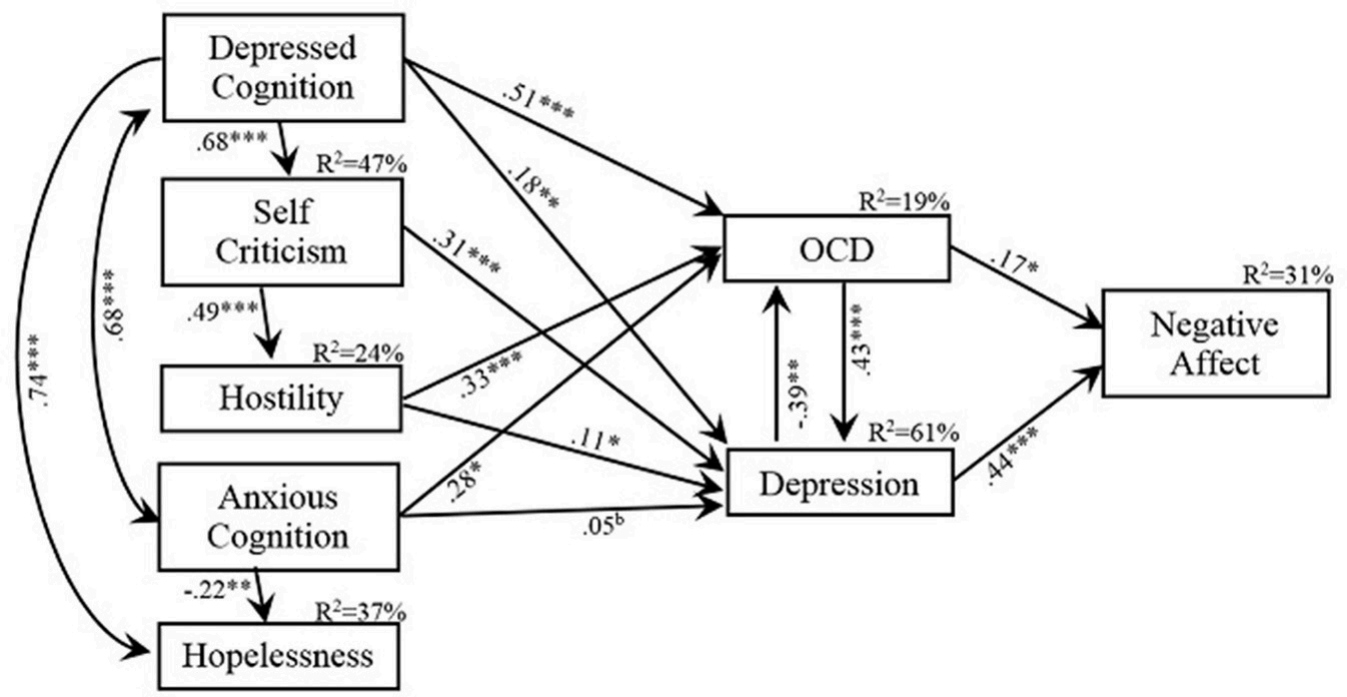
**Final model showing the standardized beta-weights for variables predictive of OCD and Depression, the relationship between OCD and Depression, their relationship with Negative Affectivity and the variance explained in OCD, Depression and Negativity Affect by their significant predictors**. ^*^p < 0.05; ^**^p < 0.01, ^***^p < 0.001, *d* = *p* > 0.05 < 0.10.

The variables in the model explained 19% of the variance in OCD, 61% in depression, and 31% in negative affectivity. Depression and OCD were significantly positively related to negative affectivity although the path from depression was stronger. OCD was positively predictive of depression while depression was negatively predictive of OCD (Figure 2).

The major standardized total effects (direct plus indirect effects) in the model indicate that OCD contributed to depression ß = 0.371 and contributed ß = 0.308 to negative affectivity while the total effect of depression on OCD was negative ß = −0.332 but positive on negative affectivity, ß = 0.325 (Table 3). Depressed cognitions had significant total effects on all variables to which they contributed, ranging from ß = 0.331 for negative affectivity to ß = 0.740 for hopelessness. Anxious cognitions had total positive effects on both depression and OCD but a negative effect on hopelessness (ß = 0.143, −220, and −0.224, respectively). The major contributions of self-criticism were to hostility and depression (ß = 0.486 and 0.370, respectively). Hostility contributed to both depression and OCD (ß = 0.219 and 0.249, respectively) and to a lesser extent to negative affectivity, ß = 0.139.

**Table 3:** Standardized total effects of ^*^variables in the final model.

	**Dep cognitions**	**Criticism self**	**Hostility**	**Anxious cognitions**	**Depression**	**OCD**
Criticism self	0.684	0.000	0.000	0.000	0.000	0.000
Hostility	0.332	0.486	0.000	0.000	0.000	0.000
Depression	0.597	0.370	0.219	0.143	−0.144	0.371
OCD	0.390	0.019	0.249	0.220	−0.332	−0.144
Negative affect	0.331	0.168	0.139	0.100	0.325	0.308
Hopelessness	0.740	0.000	0.000	−0.224	0.000	0.000

* *Standardized Total Effects are the sum of the direct effects and the product of the indirect effects*.

## Discussion

The aim of this study was to determine the relationship between scores on the symptoms of OCD and depression, and to test a model with anxious and depressive cognitions, self-criticism, hostility and hopelessness as common predictors of OCD and depression and, determine whether OCD and depression are subsumed by a common factor: negative affectivity.

As hypothesized the scores for OCD and depression are strongly related which supports the findings of van Oppen et al. (1995) and Taylor (1998) and, despite their discrete diagnostic classifications, provides some level of support for Tiller's (1994) claim that OCD is a mood disorder.

In order to explore Tiller's premise further, we presented a model to test the commonality of predictors of OCD and depression while at the same time, testing Brown et al.'s (1997) proposition that both these disorders are grounded in negative affectivity. The hypothesized model was not supported by the data however, a revised model based on theoretically sound modifications was supported. The variables in the model explained 61% of the variance in depression, 19% of the variance in OCD, and 31% of the variance in negative affectivity. Unlike the positive bivariate correlation between OCD and depression, the path from OCD to depression was positive but the path from depression to OCD was negative. This last refutes Tiller's claim that OCD is a mood disorder.

The hypothesis that there would be common predictors of OCD and depression was partially supported. Depressive cognitions and hostility predicted both OCD and depression, while anxious cognitions predicted symptoms of OCD and were in the doubtful range in predicting depression. Self-criticism was only predictive of depressive symptoms while hopelessness failed to load onto either depressive or OCD symptoms. The beta weights of the common predictors revealed a divergent pattern with anxious cognitions, depressive cognitions, and hostility loading more heavily onto OCD than depression, while self-criticism only predicted depression. That self-criticism is related to depression accords with past research that has shown that people with depression often engage in harsh self-scrutiny due to their perfectionist tendencies (Luthar et al., 1995; Zuroff et al., 1995; Fichman et al., 1996). While it is possible that the performance of compulsions can fail to decrease anxiety (Beech, 1974) and lead to an increased sense of responsibility which Salkovskis (1985,1989) argued resulted in self-critical appraisals, this was not the case in the current study.

Both anxious and depressive cognitions are related to OCD, but only depressive cognitions are related to depression: the impact of anxious cognitions of depression is borderline. These findings lend partial support to Beck et al.'s (1987) cognitive content specificity hypothesis in which they proposed that anxiotypic and depressogenic cognitions are related to anxiety disorders and depression, respectively. An explanation for the inconsistent relationship between depressive cognitions and OCD in terms of the cognitive content specificity hypothesis may be that in some people with OCD symptoms, anxiotypic thoughts related to harm and danger (e.g., “x will happen if I don't check”) coupled with repeated unsuccessful efforts to dispel anxiety through rituals (e.g., checked many times), depressive thoughts involving loss and failure (e.g., I have lost everything) may result. Or it may be that the depressive cognitions are an indication of the level of depressive co-morbidity in people with OCD.

Interestingly, hopelessness did not predict either depression or OCD. This is an unexpected finding in view of the vast amount of literature which has proposed hopelessness as a significant risk factor for depression (Kendall and Watson, 1990; Ostrander et al., 1995). It is important to note that hopelessness itself was predicted by depressive cognitions, a path that was added into the final model, and inclusion of this path in the original model may have strengthened its theoretical and clinical relevance.

Hostility was related to depression in the model which supports Kendall (1970), Moreno et al. (1993) and Selby's (1986) work, where depressed individuals showed both inner and outer directed hostility although the precise nature of the relationship was, according to Moreno et al. elusive. Consistent with Moore (2017) and Levenkron (1991) hostility was also related to OCD and this relationship was stronger than it was with depression. This finding is in contradiction to Kennedy et al. (2001) who found mixed results in terms of hostility in persons with OCD.

In terms of the total effects in the model, depressive cognitions were highly related to hopelessness, self-criticism and depression and less so to OCD, hostility, and negative affectivity. The total effect of anxious cognitions was limited to OCD and a negative effect on hopelessness. Self-criticism was positively predictive of hostility as well as depression with hostility exerting a total effect on OCD.

The hypothesis based on Brown et al.'s (1997) suggestion that OCD and depression might be grounded in negative affectivity was partially supported. As expected, both OCD and depression bivariately correlated with negative affect (*r* = 0.43 and 0.54, respectively) although the correlation between OCD and negative affect is lower than that reported by Goldberg et al. (2016) in their predominantly clinical sample. However, it must be noted that the Obsessive-Compulsive Inventory-Revised used by Goldberg et al. contains items which refer to hoarding as well as obsessions and compulsions. In the current model, it is clear that depression is a stronger indicator of negative affectivity than is OCD (ß = 0.44 and 0.17, respectively). The finding that OCD is related to depression suggests that it also has an indirect association with negative affectivity, albeit through depression. This suggestion echoes Montgomery's (1993) claim that, at least in the current study, depression is an integral part of OCD but that OCD is fundamentally an independent disorder from depression. Therefore it can be argued that there is a high co-morbidity rate of depression in OCD but less so the reverse.

It is important to note that only 31% of the variance in negative affectivity was explained by the variables in the model. It might be that stress and avoidant coping strategies are implicated in feelings of negative affectivity as well as the personality factors of neuroticism and introversion, and an external locus of control or at least, a low sense of internal control. Future research would be required to test these suggestions.

While the current study is limited by the use of a non-clinical sample, the findings do provide some clarification of the relationship between the symptoms of OCD and depression as assessed using psychometrically robust questionnaires designed to assess these traits. Although, there was a strong correlation between these constructs, the path analysis clearly indicates that they are not the same construct, neither are they both fully subsumed by negative affectivity. It is important that a future study tests this model using clinical samples to replicate the current findings as the trajectory of the disorders and diverse treatment regimes, and probable co-morbidities seen in clinical samples may not reflect the current non-clinical results. It is also important that future studies using clinical samples include other factors, such as personality, perfectionism, dependency, and autonomy which have been related to OCD, depression and negative affectivity to varying degrees. Future research should also confirm that OCD stands-alone from the anxiety disorders as indicated by its diagnostic separation in the latest edition of the DSM (DSM-V, American Psychiatric Association, 2013), particularly as the DSM acknowledges the close relationship between the anxiety disorders and OCD (*p*. 235). Research using methods such as the clinician rated Structured Clinical Interview for DSM-5 (SCID-5) may provide a further basis for establishing the validity of the relationship between OCD and depression.

In conclusion, while the current data are not from clinical samples, they do suggest a lack of support for Tiller's (1994) claim that OCD is a mood disorder: rather, the current findings provide support for their discrete classifications as in the DSM-V and as suggested by several authors (e.g., Montgomery, 1993). Neither was there support for Brown et al.'s (1997) suggestion that both disorders are subsumed by negative affectivity. While there is a relationship between OCD and depression, and some commonality of the predictors of each as tested here, clinicians need to be aware of the divergent pattern or weighting of these predictors with respect to each disorder.

## Author contributions

All authors listed, have made substantial, direct and intellectual contribution to the work, and approved it for publication.

### Conflict of interest statement

The authors declare that the research was conducted in the absence of any commercial or financial relationships that could be construed as a potential conflict of interest.
